# Sensory Preference of Drinking Water Influenced by Subthreshold‐Level Mineral Salt Mixtures

**DOI:** 10.1002/fsn3.71339

**Published:** 2025-12-11

**Authors:** Xiaowei Li, Xin Shen, Mohan Zhou, Junxi Lin, Miduo Yuan, Yan Tong, Li Chen, Shiyi Tian, Yuezhong Mao

**Affiliations:** ^1^ School of Food Science and Biotechnology Zhejiang Gongshang University Zhejiang China; ^2^ China‐UK Joint Research Laboratory of Eating Behaviour and Appetite Zhejiang Gongshang University Zhejiang China

**Keywords:** design of experiments, drinking water, mineral salts, sensory preference, subthreshold‐level

## Abstract

This study investigated the impact of subthreshold‐level mixtures of KCl and MgSO_4_ on the sensory preference of drinking water and identified key sensory drivers. Absolute thresholds were first determined, followed by sensory evaluation of individual and blended salt solutions using a 9‐point hedonic scale and CATA test. Design of Experiments (DOE) approach with quadratic polynomial regression optimized the binary mixtures. Results showed samples near the absolute thresholds (0.95 mg/L KCl; 0.65 mg/L MgSO_4_) were most preferred. Beyond these thresholds, mineral salt concentration negatively correlated with hedonic rating. Sweetness was identified as a key positive driver, while astringency was a strong negative driver. The significant model revealed a nonadditive effect with a negative KCl × MgSO_4_ interaction (antagonism), with a maximum predicted liking of 6.34 at 0.92 mg/L KCl and 0.60 mg/L MgSO_4_. These findings demonstrate that subthreshold‐level mixtures of mineral salts within a narrow concentration window can enhance water sensory quality by promoting sweetness and suppressing astringency, providing a foundation for developing premium bottled water.

## Graphical abstract

A distinct preference plateau was identified, with liking maximized near the salts' absolute thresholds. Sweetness promotion and astringency suppression were the primary sensory mechanisms driving preference in these mineral salt mixtures. A predictive model pinpointed the optimal blend (0.92 mg/L KCl; 0.60 mg/L MgSO_4_), providing a practical, data‐driven framework for superior‐tasting drinking water.

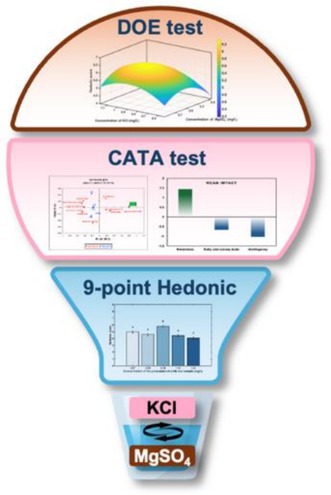

## 1 Introduction

Drinking water safety was fundamentally linked to human health. It was estimated that approximately 80% of global disease incidence was related to water pollution (Goyal et al. 2021), highlighting a close relationship between water quality and both public health and quality of life. With the high‐quality development of the social economy and the significant improvement in living standards, consumer demand for drinking water shifted from merely quenching thirst to pursuing high‐quality sensory attributes, including taste, flavor, and overall drinking experience (Shuai et al. 2024). Recent consumer studies further show that organoleptic judgments (taste/odor) and safety perceptions strongly shape bottled‐versus tap‐water choices. In US national data (*n*≈4000), adults who rated local tap water taste poorly or believed bottled water was safer had markedly higher odds of drinking bottled water and lower odds of drinking tap water (Park et al. 2023). Against this backdrop, research on and evaluation of the sensory quality of drinking water became essential. It not only served as a critical foundation for meeting consumer preferences but also acted as an important means to promote the upgrading of the drinking water industry and safeguard public health.

Early research on drinking water was predominantly concentrated on physicochemical water quality within the domain of aquatic environmental safety. Horton (1965) pioneered the development of a Water Quality Index (WQI), which established a theoretical foundation for subsequent diverse water quality assessment frameworks. This index integrated multiple physicochemical parameters and converted them into a single composite score or classification, thereby substantially mitigating subjective bias in evaluations. It systematically assessed water quality based on nine key parameters: total biochemical oxygen demand, dissolved oxygen, coliform density, pH, temperature, total nitrate, total phosphate, total dissolved solids (TDS), and turbidity.

Building upon this foundation, Ramesh et al. (2010) formulated a Drinking Water Quality Index (DWQI) to systematically evaluate the suitability of groundwater resources for potable purposes. Their model incorporated 22 core parameters, including physicochemical indicators, trace metals, inorganic pollutants and microbiological indicators. The DWQI quantified these parameters into standardized scores and aggregated them into a composite value using a weighted model. The study concluded that the geometric mean‐based aggregation model offered a more accurate representation of actual water quality compared to the arithmetic mean method. This was primarily because the geometric mean is less sensitive to extreme values, aligning better with the typically log‐normal distribution of water quality parameters.

While these indices facilitated objective water quality assessment and significantly reduced human‐introduced systematic error, a significant disconnect remained with consumers' direct sensory experiences. Parameters such as electrical conductivity and concentrations of metal ions proved difficult to correlate directly with taste attributes like sweetness, astringency, or smoothness.

With the continuous growth in bottled water consumption, a systematic investigation of consumers' sensory preferences has become increasingly urgent. The water quality parameters influencing consumer sensory experience can be primarily categorized into source water type, pH, metal ions, and other factors. Significant correlations (Sipos et al. 2012) were identified among key physicochemical parameters: TDS showed strong positive correlations with HCO₃^−^, Ca^2+^, and Mg^2+^, while pH was positively correlated with salty, sweet, and bitter tastes. From the perspective of a sensory evaluation index system, the factors influencing the sensory properties of drinking water were systematically categorized into two major classes: organic and inorganic factors. Organic factors typically referred to organic substances derived from microbial metabolism or anthropogenic discharges, including geosmin, mercaptans, trihalomethanes, β‐cyclocitral, and 2‐methylisoborneol (Adams et al. 2025; Otten et al. 2016; Shang et al. 2024). These compounds primarily affected the sensory characteristics of water by inducing distinct off‐flavors, which were described as earthy/musty, sulfurous, medicinal, or woody (Shi et al. 2022). Some substances could even produce more intense odors, such as tar‐like, rotten egg, or chlorobenzene‐like smells (Kitajima et al. 2025; Zaitlin and Watson 2006).

Furthermore, studies (Armenteros et al. 2012; Lawless et al. 2003) indicated that divalent cation salts, such as calcium chloride, magnesium chloride, and MgSO_4_, primarily imparted a bitter taste, accompanied by secondary taste sensations of saltiness, metallic notes, astringency, and sourness, with the intensity of these sensations generally following a decreasing trend. Tordoff 1996 investigated the taste profiles of calcium salts at suprathreshold concentrations. They reported that as the concentration increased, the sweetness of calcium chloride solutions decreased while their saltiness intensified (Tordoff 1996). At equimolar concentrations, calcium chloride solutions exhibited a stronger bitter taste and a slightly weaker sour taste compared to calcium lactate solutions. Notably, while suprathreshold concentrations often triggered irritating sensations, metal ions at subliminal concentrations demonstrated flavor‐modulating properties. For instance, iron was involved in polyphenol oxidation and the generation of flavor compounds (Nahar and Zhang 2013).

Existing research has predominantly focused on individual components. However, the human sensory system inherently perceives flavor as an integrated response to multiple components acting synergistically. A study by Baryłko‐Pikielna and Kostyra (2007) demonstrated that the combination of monosodium glutamate with trace nucleotides not only significantly enhanced umami perception but also augmented the overall food flavor and improved consumer hedonic ratings. This finding revealed a synergistic enhancement mechanism of compound flavor substances on food sensory attributes. While this synergistic mechanism has been extensively validated in complex food matrices, research in the context of drinking water remains insufficient. This gap, to some extent, has hindered the advancement of the sensory quality of drinking water.

**FIGURE 1:**
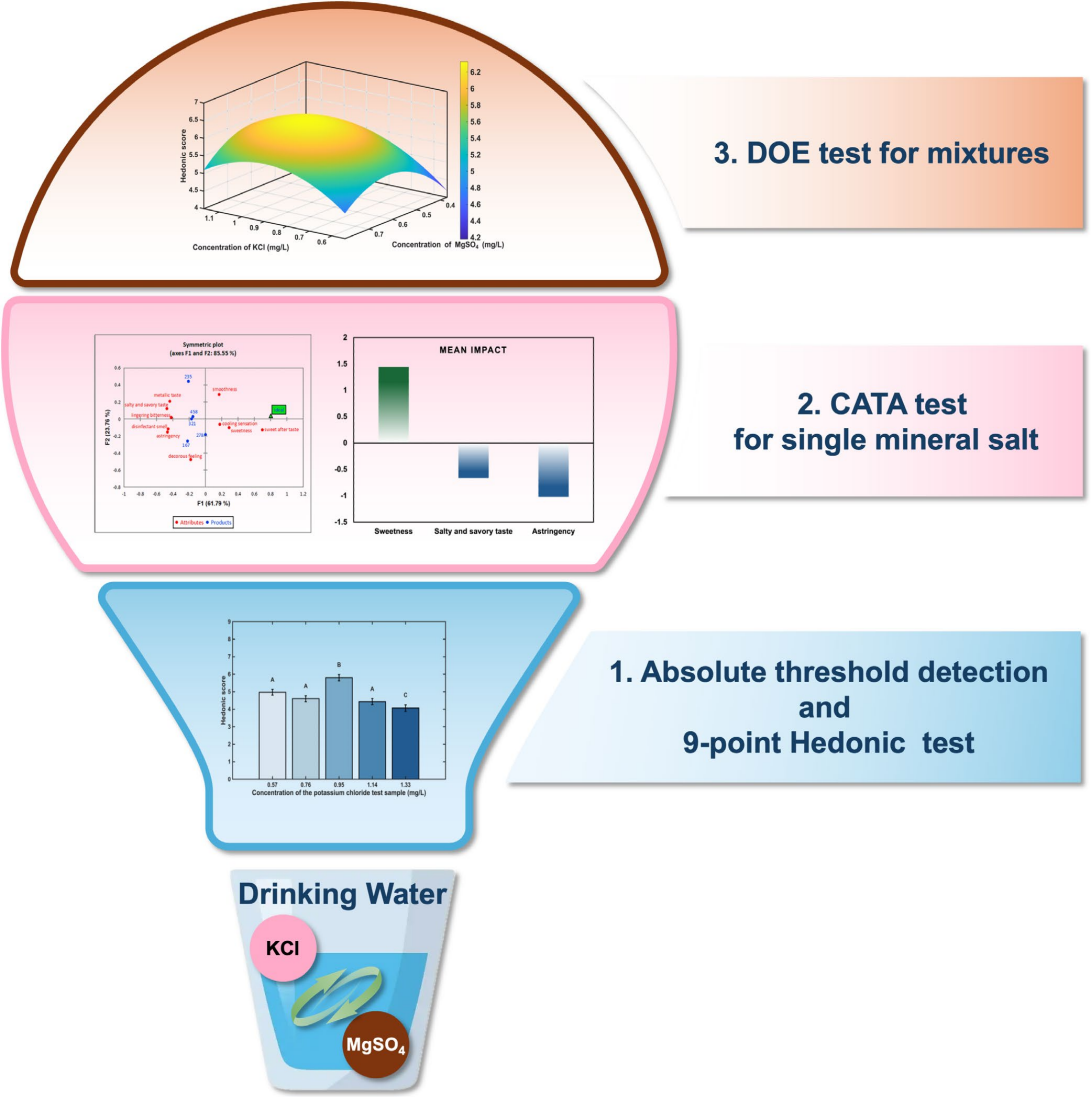
Experiment design of this study.

Therefore, this study aimed to investigate the impact of subthreshold and near‐threshold levels mineral salt mixtures on the sensory preference of drinking water. A systematic approach was employed to achieve this objective. First, the absolute thresholds of the target mineral salts were determined using the triangle test method. Subsequently, scaling method and Check‐All‐That‐Apply (CATA) tests were utilized to identify the sensory characteristics and the optimal preferred concentration of aqueous solutions containing individual mineral salts. Finally, a Design of Experiments (DOE) approach was applied to study the effects of combining these mineral salts within their subthreshold concentration ranges on the sensory hedonic perception of drinking water (as shown in Figure 1). This study provided valuable insights into the mechanisms of human perception toward subthreshold and near‐threshold mixtures of mineral salts, thereby paving the way for the development of drinking water with superior sensory quality.

## 2 Materials and Methods

### 2.1 Materials

In accordance with the Chinese National Food Safety Standard GB 2760‐2024, “Standard for Uses of Food Additives”, the mineral salts permitted in drinking water and their maximum usage levels are MgSO_4_·7H_2_O (50 mg/L), ZnSO_4_ (6 mg/L), and calcium chloride (100 mg/L), with KCl permitted for use as needed. Based on preliminary experiments, MgSO_4_·7H_2_O (in the following text, it was simplified as MgSO_4_) and KCl were selected as the target mineral salts for this investigation. MgSO_4_ and KCl were procured from Merck Chemical Technology (Shanghai) Co. Ltd. The purified water (pH = 7) used in the experiments was supplied by Hangzhou Wahaha Group Co. Ltd.

Test samples were prepared and evaluated at room temperature, with each assessor receiving a 30 mL portion per sample to ensure consistency in serving size and sensory exposure.

### 2.2 Sensory Panel

The sensory panel comprised 30 selected assessors (15 males and 15 females, aged 20–35 years), recruited in accordance with the Chinese national standard GB/T 16291.1‐2012 Sensory analysis—General guidance for the selection, training, and monitoring of assessors—Part 1: Selected assessors. Each assessor was informed of the study objectives and completed a two‐week training program, conducted once daily. The training included the following components:

(1) Sensory evaluation terminology: Assessors received instruction on terms such as attribute and hedonic.

(2) Basic taste discrimination: Assessors were required to correctly identify and differentiate basic taste sensations.

(3) Methodological training: triangle test, CATA and scaling method procedures were introduced and practiced.

The 30 assessors will participate in all sensory evaluation sessions of the study within a professional sensory evaluation laboratory, ensuring standardized testing conditions. In addition, assessors were instructed to refrain from smoking or eating for at least 30 min before each evaluation session to minimize potential interferences.

### 2.3 Determination of the Absolute Threshold for Mineral Salts

The absolute threshold detection method for mineral salts was based on our previous study (Mao et al. 2022). Based on the preliminary experiments, a series of concentrations for MgSO_4_ (0.50, 0.60, 0.70, 0.80 mg/L) and KCl (0.80, 0.90, 1.00, 1.10 mg/L) were prepared in aqueous solution. Taking the MgSO_4_ as an example, the absolute threshold detection procedure was as follows:

Step 1: Setting 5 different concentrations of MgSO_4_ solutions as absolute threshold samples. Marking three random numbers on each one of the three identical test cups. Taking MgSO_4_'s absolute threshold sample 1 as the test object and pouring 20 mL of sample 1 into one or two of the three marked cups. Then, pouring 20 mL of the compared sample (ultrapure water) into the left two cups or one cup. The above three cups of samples composed one test group. Preparing 30 same test groups and naming them as test round 1.

Step 2: The MgSO_4_'s absolute threshold samples 2 to 5 were used as the test object, respectively. Then, repeat Step 1 and prepare the test rounds 2 to 5.

Step 3: The sensory panel was asked to sit in the sensory evaluation room. Each assessor sat in a separate seat and was given one test group for test round 1.

Step 4: Assessors were instructed to rinse their mouths with 20 mL ultrapure water for 10 s and then expectorate.

Step 5: Each assessor was asked to take one cup of the test group and hold it in the mouth for 10 s and then expectorate. After that, they were instructed to rinse their mouths with 20 mL ultrapure water for 10 s and then expectorate.

Step 6: Each assessor was asked to repeat steps 4 and 5 to evaluate the left two cups of the test group. Then, each assessor was asked to write down the different one's three random numbers on the answer sheet.

Step 7: Each assessor was asked to repeat steps 4–6 to evaluate test rounds 2 to 5 and to write down each test round's different one's three random numbers.

Step 8: Checking the answer of each test round. If one test round's correct option number was less than 15, this test round's evaluation result was wrong. If one test round's correct option number was equal to or greater than 15, this test round's evaluation result was right. Thus, five test rounds' evaluation results could be obtained.

Step 9: If the test round *n*'s evaluation result was wrong, and all the followed test rounds' evaluation results were right at the same time. The MgSO_4_'s absolute threshold value could be calculated according to the following equation:

$$C_{M.\mathrm{atv}}=\sqrt{C_{\text{atvn}}\times C_{\text{atvn}+1}}$$

where $C_{\text{atvn}}$ was the sample concentration of test round *n*, $C_{\text{atvn}+1}$ was the sample concentration of the test round after round *n*.

### 2.4 9‐Point Hedonic Test for Single Mineral Salts Aqueous Solution

This study investigated consumer preference for drinking water with varying concentrations of MgSO_4_ and KCl using a 9‐point hedonic scale (1 = “dislike extremely”, 2 = “dislike very much”, 3 = “dislike moderately”, 4 = “dislike slightly”, 5 = “neither like nor dislike”, 6 = “like slightly”, 7 = “like moderately”, 8 = “like very much”, 9 = “like extremely”). For each compound, five concentration levels were prepared. These levels were established based on the predetermined absolute threshold of each mineral salt, creating a series that included 60%, 80%, 100%, 120%, and 140% of this threshold value.

### 2.5 CATA Test for Single Mineral Salts Aqueous Solution

To investigate the impact of MgSO_4_ and KCl on the sensory attributes of drinking water, the Check‐All‐That‐Apply (CATA) method was employed. Aqueous solutions of each compound were evaluated at five concentration levels, identical to those described in Section 2.4. The lexicon of sensory attributes used in the CATA test included: sweetness, salty and savory taste, astringency, metallic taste, disinfectant smell, bitter aftertaste, sweet aftertaste, cooling sensation, mouthfeel, and smoothness.

### 2.6 DOE Test for Mixtures of Mineral Salts Aqueous Solution

Design of Experiments (DOE) is a systematic methodology grounded in statistical principles, used to analyze the effects of multiple factors on a target response (Mao et al. 2024). Building upon the hedonic ratings obtained from the single mineral salt solutions, a DOE was employed to investigate the preference for drinking water containing subthreshold‐level mineral salt mixtures. KCl concentration and MgSO_4_ concentration were selected as the two factors, each with four levels. The 9‐point hedonic score served as the response variable. The specific experimental design was presented in Table 1.

**TABLE 1:** Subthreshold‐level mineral salt mixtures for DOE test.

Compound	Series number of test mixtures and its concentration (mg/L)															
	1	2	3	4	5	6	7	8	9	10	11	12	13	14	15	16
KCl	0.57	1.14	1.14	1.14	0.57	0.95	0.76	0.76	0.95	0.76	0.76	0.57	0.95	0.95	1.14	0.57
MgSO_4_	0.78	0.52	0.78	0.65	0.52	0.39	0.53	0.78	0.65	0.65	0.39	0.39	0.78	0.52	0.39	0.65

## 3 Results and Discussion

### 3.1 Absolute Threshold of Mineral Salts

Based on the sensory panel test results and previous findings from our laboratory (Mao et al. 2019), the absolute threshold for KCl in purified water was determined to be 0.95 mg/L, calculated from the intermediate concentration between the incorrectly identified concentration (0.90 mg/L) and the correctly identified concentration (1.0 mg/L). Similarly, the absolute threshold for MgSO_4_ was established at 0.65 mg/L, derived from the midpoint between its incorrectly identified concentration (0.60 mg/L) and correctly identified concentration (0.70 mg/L).

### 3.2 9‐Point Hedonic Test Results of Single Mineral Salts Aqueous Solution

**FIGURE 2:**
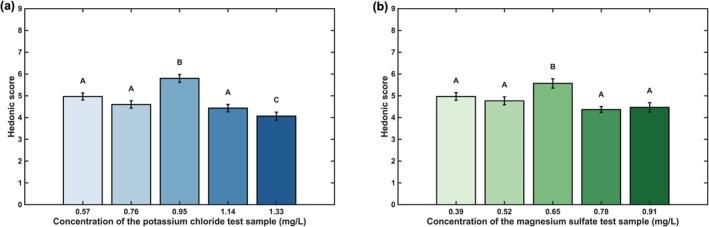
9‐point hedonic test results of: (a) KCl, (b) MgSO_4_.

Based on the absolute threshold results, the test concentrations for KCl in the 9‐point hedonic test were set at 0.57, 0.76, 0.95, 1.14, and 1.33 mg/L, and for MgSO_4_ at 0.39, 0.52, 0.65, 0.78, and 0.91 mg/L, respectively. The detailed 9‐point hedonic test results were shown in Figure 2. As shown in Figure 2a, based on multiple comparisons analysis (Tukey–Kramer), the five drinking water samples with varying KCl concentrations were categorized into three distinct statistical groups according to their hedonic scores. Samples containing 0.57, 0.76, and 1.14 mg/L of KCl were grouped together. The sample with 0.95 mg/L KCl formed a separate group, which received the highest mean hedonic score of 5.80. The sample with the highest concentration (1.33 mg/L) constituted the third group and received the lowest preference score. The complete results of the hedonic ratings for the KCl samples are presented in Figure 2b. A similar analysis for MgSO_4_ revealed that the sample with a concentration of 0.65 mg/L formed a unique group and achieved the highest hedonic score of 5.57. The remaining four concentrations were grouped together, with significantly lower preference scores.

Collectively, these results indicated that for both KCl and MgSO_4_, the samples prepared at their respective absolute threshold concentrations (0.95 mg/L for KCl and 0.65 mg/L for MgSO_4_) were the most preferred. Concentrations deviating from this threshold, particularly those significantly exceeding it, resulted in a marked decrease in consumer liking. This suggests a negative correlation between mineral salt concentration and hedonic rating once the absolute threshold is surpassed.

### 3.3 CATA Test Results of Single Mineral Salts Aqueous Solution

**FIGURE 3:**
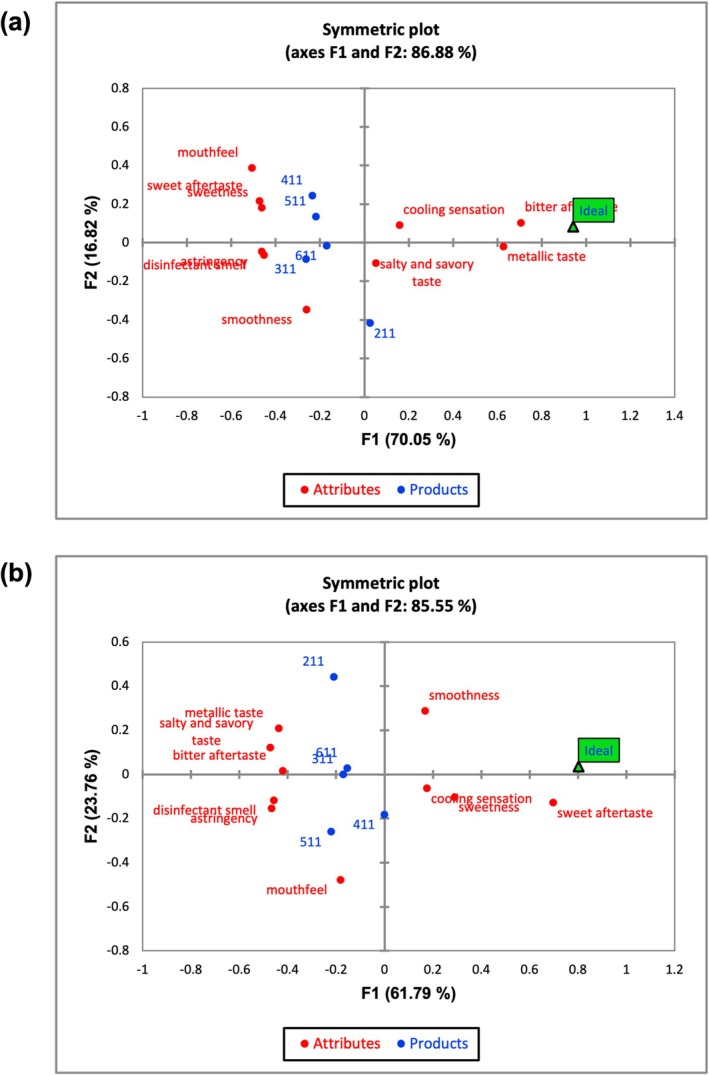
CATA test results of: (a) KCl, (b) MgSO_4_.

**FIGURE 4:**
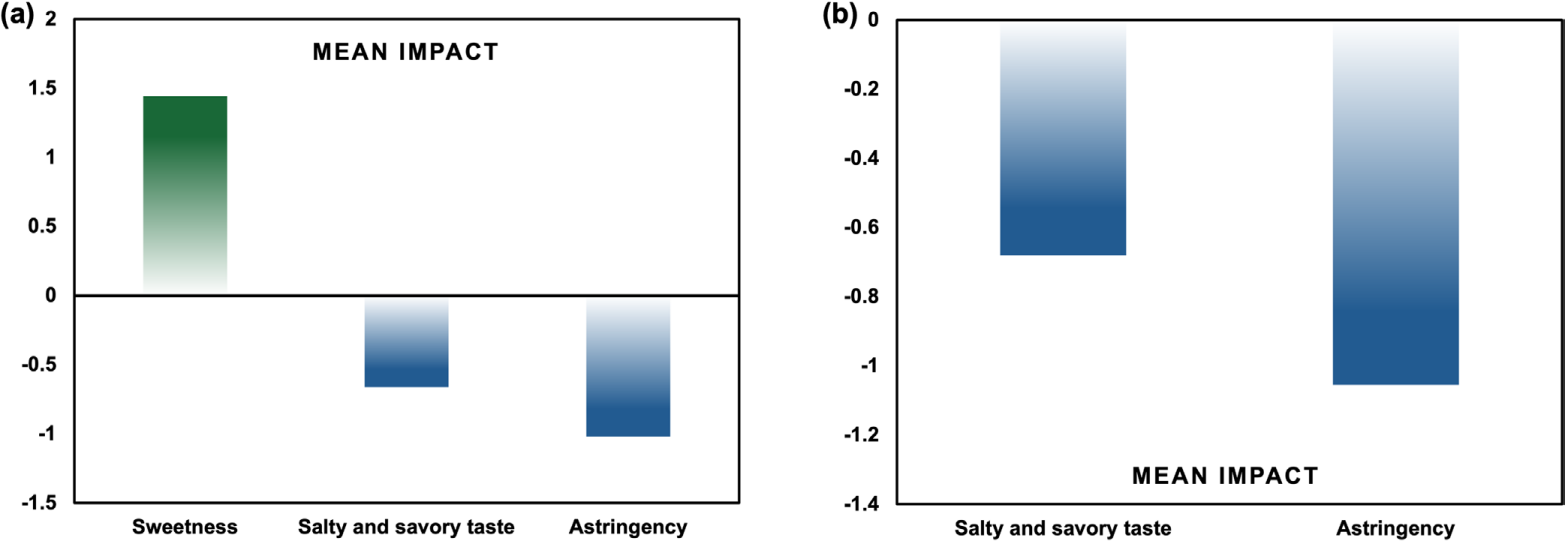
Mean impact results of: (a) KCl, (b) MgSO_4_.

The CATA results for drinking water samples with varying concentrations of KCl and MgSO_4_ are presented in Table 2, Figure 3a,b, respectively. Analysis of the frequency of term selection revealed that “cooling sensation” and “smoothness” were the most frequently selected descriptors. Attributes such as “mouthfeel”, “salty and savory taste”, “sweet aftertaste”, “metallic taste”, and “sweetness” were selected with moderate frequency. In contrast, “astringency”, “bitter aftertaste”, and “disinfectant smell” were among the least selected terms. Meanwhile, the drinking water samples with different KCl concentrations showed significant differences in the attributes of smoothness and mouthfeel. Similarly, significant differences in smoothness were observed among the samples with varying MgSO_4_ concentrations. Figure 4a,b presents the mean impact of sensory attributes from the CATA test for drinking water supplemented with KCl and MgSO_4_, respectively. Comparative analysis revealed that sweetness (mean impact value = 1.441) emerged as a key positive driver of liking. In contrast, salty and savory taste and astringency (mean impact value = −0.662, −1.02 in KCl and −0.68, −1.055 in MgSO_4_) were identified as negative drivers, with astringency exhibiting a particularly strong negative impact. Although MgSO_4_ was typically linked to bitterness, our CATA showed that “astringency” had a strong negative mean impact. We interpreted this as a perceptual compound, not a discrete astringent mechanism: (i) frequent co‐selection with bitterness/metallic inflated the penalty; (ii) in a low‐flavor water matrix, modest increases in ionic strength/sulfate reduced oral lubrication and prolonged a bitter/metallic afterfeel described as “drying”; and (iii) KCl plus MgSO_4_ accentuated this adverse complex. Thus, we treated “astringency” as a consumer‐facing summary of a bitter‐metallic‐drying mouthfeel, not intrinsic astringency of MgSO_4_. These findings indicate that sweetness and astringency are critical determinants in the sensory preference of drinking water, serving as principal pathways through which these metal salts modulate its overall sensory quality.

**TABLE 2:** Cochran's *Q* test result of CATA for KCl and MgSO_4_.

Attributes	*p*	
	KCl	MgSO_4_
Sweetness	0.625	0.174
Salty and savory taste	0.174	0.720
Astringency	0.126	0.126
Metallic taste	0.150	0.578
Disinfectant smell	0.856	0.896
Bitter aftertaste	0.373	0.887
Sweet aftertaste	0.896	0.373
Cooling sensation	0.007	0.150
Mouthfeel	0.736	0.053
Smoothness	0.027	0.015

Cross‐referencing these findings with the 9‐point hedonic ratings indicated a clear association between specific attributes and consumer preference. Samples with higher liking scores were predominantly characterized by sweetness, cooling sensation, and smoothness. Conversely, samples with lower preference were often linked to metallic taste, disinfectant smell, and astringency. Overall, the sensory profile of the most preferred drinking water samples was defined by the presence of sweetness, cooling sensation, smoothness, and a sweet aftertaste.

### 3.4 DOE Test Results for Mixtures of Mineral Salts Aqueous Solution

The hedonic data from the 9‐point scale evaluation of 16 drinking water samples with varying concentrations of KCl and MgSO_4_ (set by DOE) were analyzed using a quadratic polynomial regression analysis in MATLAB. The following second‐order regression model was established:

$$Y=-7.30+14.19\times K+23.58\times M-7.34\times K^{2}-1.15K\times M-18.64\times M^{2}$$

where *Y* represents the hedonic score, *K* denotes the concentration of KCl (mg/L), and *M* signifies the concentration of MgSO_4_ (mg/L).

Furthermore, ANOVA confirmed that the model relating hedonic preference to the concentrations of KCl and MgSO_4_ was statistically significant (*p* < 0.05). This significance suggests that the two mineral salts, within their subthreshold concentration ranges, exhibited a nonadditive effect. The fitted cross‐term (KCl × MgSO_4_) was negative, indicating antagonism within the subthreshold design space. The observed “high‐score plateau” and the predicted optimum (6.34 at 0.92 mg/L KCl and 0.60 mg/L MgSO_4_) arose from the significant curvature (quadratic) terms and optimal main‐effect levels rather than a positive (synergistic) cross‐term.

**FIGURE 5:**
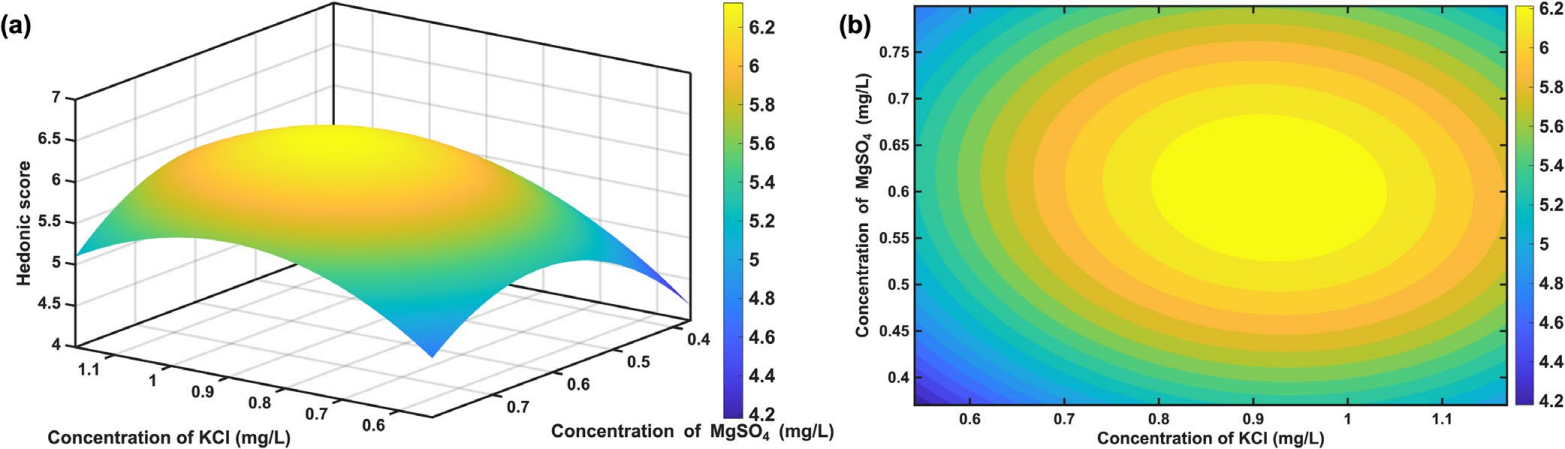
Response surface (a) and contour plots (b) of KCl and MgSO_4_ mixtures.

The response surface and contour plots generated based on this model (as shown in Figure 5a,b, respectively) clearly illustrated the pattern of sensory preference variation. As shown in the figures, a distinct “plateau” of high hedonic scores was identified, corresponding to a specific combination range of KCl and MgSO_4_ concentrations. When the formulation fell within this optimal region, the hedonic score of the drinking water could be maintained at a high level (greater than 6.0). Specifically, the model predicted the optimal conditions for hedonic preference. The maximum score of 6.34 was achieved at KCl and MgSO_4_ concentrations of 0.92 and 0.60 mg/L, respectively.

Mechanistically, the preference peak we observed near the absolute thresholds of KCl and MgSO_4_, followed by a rapid drop as concentrations deviated (especially upward), aligns with prior human studies on mineralized waters. Those studies reported “high‐score plateaus” and steep declines on response surfaces when multiple ions were present—that is, a narrow optimal window where liking is high, but once concentrations rise beyond it, bitter, metallic, and astringent notes emerge, and acceptance falls. This matches the “plateau to decline” pattern in our surface and supports a practical takeaway: formulations tuned close to absolute thresholds are more acceptable to consumers (Bruvold and Gaffey 1969; Lim and Lawless 2005). Within this threshold‐adjacent region, the observed KCl‐MgSO_4_ interaction may tentatively be interpreted not as the emergence of a qualitatively new pleasant flavor, but as a partial dampening of bitterness, metallic notes and drying mouthfeel. More detailed work using quantitative descriptive analysis (QDA) across the same concentration range will be required to verify whether mutual suppression of specific negative attributes is indeed the main pathway by which liking is maintained at a high level.

Regarding sensory drivers, our data indicate that sweetness was a positive driver of liking, whereas astringency and signals associated with salty/savory taste were negative, with astringency especially impactful. This is consistent with taste‐interaction work showing that modest sweetness can attenuate KCl's bitter‐metallic character and improve acceptability, yet divalent salts such as MgSO_4_ readily introduce bitter/metallic notes and astringency that suppress overall liking. Practically, keeping KCl and MgSO_4_ within a threshold‐adjacent “cooperative band” preserves a sweetness advantage and prevents negative attributes from dominating, which explains both the stable > 6.0 region on our response surface and the predicted optimum of 6.34 (Zhu et al. 2020).

## 4 Conclusions

Our findings demonstrate that consumer preference for mineralized water peaked when KCl and MgSO_4_ were formulated at, or just below, their absolute thresholds (0.95 and 0.65 mg/L, respectively). Departures from these levels‐particularly higher concentrations‐produced marked decreases in liking, consistent with the emergence of bitter/metallic notes and astringency. Response surface and contour analyses mapped a narrow, threshold‐adjacent “plateau” where hedonic scores were stably high (> 6.0); within this region the model predicted a maximum liking of 6.34 at 0.92 mg/L KCl and 0.60 mg/L MgSO_4_. ANOVA confirmed model significance (*p* < 0.05) and supported the presence of interaction between the two salts in the subthreshold regime, indicating that their effects were not simply additive.

Driver analysis clarified the sensory levers for optimization: sweetness contributed positively to acceptance, while astringency and salty/savory signals were negative drivers, with astringency exerting the strongest penalty. From a formulation standpoint, these results recommend controlling KCl and MgSO_4_ within a tight, threshold‐adjacent (±0.18 and 0.12 mg/L, respectively) band to preserve sweetness benefits and avoid the rapid rise of negative attributes. Practically, this guidance can support target setting for mineral dosing, validation of supplier specifications, and quality‐by‐design limits in bottled or point‐of‐use waters.

Limitations include the focus on two salts and a single product matrix. It should be emphasized that the present response surface model characterizes preference dynamics only within a narrow concentration window around the absolute thresholds of KCl and MgSO_4_. Any extrapolation of this threshold‐adjacent pattern to deeper subthreshold regions should therefore be made cautiously. Future work should test generalization across broader ionic compositions, pH and temperature conditions, and consumer groups, and evaluate mitigation strategies (e.g., sweetness modulation) to maintain acceptance when higher mineralization is unavoidable.

## Author Contributions

**Xiaowei Li:** methodology, software, formal analysis, data curation, writing – original draft, investigation. **Xin Shen:** methodology, investigation, formal analysis, data curation. **Mohan Zhou:** methodology, investigation, software, data curation. **Junxi Lin:** investigation, methodology, software. **Miduo Yuan:** methodology, investigation, formal analysis. **Yan Tong:** methodology, software. **Li Chen:** validation, project administration, writing – review and editing. **Shiyi Tian:** funding acquisition, writing – review and editing, visualization, project administration, supervision.**Yuezhong Mao:** conceptualization, resources, investigation, funding acquisition, writing – review and editing.

## Funding

This work was supported by the Zhejiang Provincial Natural Science Foundation of China (LTGN23C200006).

## Ethics Statement

The whole study was approved by the Ethics Committee of Zhejiang Gongshang University (No. 24134704). All participants were asked to provide informed consent prior to participation, with explicit information about data confidentiality and voluntary withdrawal rights.

## Conflicts of Interest

The authors declare no conflicts of interest.

## Data Availability

The data that support the findings of this study are available on request from the corresponding author. The data are not publicly available due to privacy or ethical restrictions.
